# Prospective clinical sequencing of 1016 Chinese prostate cancer patients: uncovering genomic characterization and race disparity

**DOI:** 10.1002/1878-0261.13511

**Published:** 2023-08-23

**Authors:** Yu Wei, Tingwei Zhang, Beihe Wang, Jian Pan, Shengming Jin, Bangwei Fang, Weijie Gu, Xiaojian Qin, Bo Dai, Guowen Lin, Hualei Gan, Junlong Wu, Dingwei Ye, Yao Zhu

**Affiliations:** ^1^ Department of Urology Fudan University Shanghai Cancer Center China; ^2^ Department of Oncology, Shanghai Medical College Fudan University Shanghai China; ^3^ Shanghai Genitourinary Cancer Institute China; ^4^ Department of Pathology Fudan University Shanghai Cancer Center China

**Keywords:** Chinese population, clinical sequencing cohort, ethnic disparity, *FOXA1*, prostate cancer

## Abstract

Although there is a well‐known disparity in prostate cancer (PC) incidence and mortality between Chinese and Western patients, the underlying genomic differences have been investigated only sparsely. This clinicogenomic study was conducted to reveal the genomic mutations contributing to the PC disparity across ethnicities and investigate the mutational profile of Chinese PC patients. A total of 1016 Chinese PC patients were prospectively enrolled and subjected to targeted sequencing, resulting in usable sequencing data for 41 genes from 859 patients. Genomic data retrieved from The Cancer Genome Atlas (TCGA; locoregional PC), Memorial Sloan Kettering Cancer Center [MSKCC; metastatic castration‐sensitive PC (mCSPC)], and Stand Up To Cancer [SU2C; metastatic castration‐resistant PC (mCRPC)] cohorts were used as comparators representing Western men. Genomic mutations were analyzed using an integrated bioinformatic strategy. A comparison of the disease stages revealed that mutations in tumor protein 53 (*TP53*), androgen receptor (*AR*), forkhead box A1 (*FOXA1*), and genes involved in the cell cycle pathway were enriched in mCRPC. Mutations in adenomatous polyposis coli (*APC*) gene were found to be more prevalent in patients with visceral metastasis. Genomic differences between Western and Chinese men were mainly observed in castration‐sensitive PC, with tumors from Chinese men having more *FOXA1* (11.4% vs. 4.2%) but fewer *TP53* (4.8% vs. 13%) mutations in locoregional PC and harboring fewer *TP53* (11% vs. 29.2%), phosphatase and tensin homolog (*PTEN*; 2.5% vs. 10.3%), and *APC* (1.7% vs. 7.4%) mutations in the mCSPC stage than those of Western men. Patients of both ethnicities with mCRPC had similar mutational spectra. Furthermore, *FOXA1* class‐2 was less common than *FOXA1* class‐1 and showed no enrichment in metastasis, contrary to the findings in the Western cohort. Our study provides a valuable resource for a better understanding of PC in China and reveals the genomic alterations associated with PC disparity across races.

## Graphical abstract

The authors sequenced the genomic DNA from 1016 Chinese prostate cancer patients. Mutations in *TP53*, *AR*, and *FOXA1* were enriched in metastatic castration‐resistant prostate cancers (mCRPCs), and *APC* mutations were enriched in liver metastasis. The authors also found notable genomic difference between tumors in Chinese and Western men, with more *FOXA1* but fewer *TP53*, *PTEN*, and *APC* mutations in Chinese men.

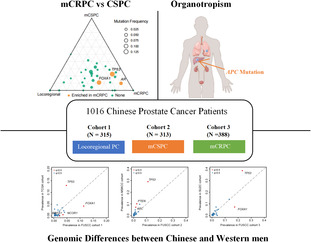

**Abbreviations**
ADT: androgen deprivation therapy
ARSI: androgen receptor signaling inhibitors
ASR: age‐standardized rate
CPGEA: Chinese Prostate Cancer Genome and Epigenome Atlas
ctDNA: circulating tumor DNA
DDR: DNA damage repair
FUSCC: Fudan University Shanghai Cancer Center
ISUP: International Society of Urological Pathology
MAF: mutant allele frequency
mCRPC: metastatic castration‐resistant prostate cancer
mCSPC: metastatic castration‐sensitive prostate cancer
PARP: poly (ADP‐ribose) polymerase
PCa: prostate cancer
rPFS: radiographic progression‐free survival
SEER: Surveillance, Epidemiology, and End Results Program
VAF: variant allele frequency

## 1 Introduction

Prostate cancer (PC) is the second most frequent malignancy among men worldwide but displays great diversity in incidence and mortality across regions and ethnicities [1, 2]. According to the GLOBOCAN report, the age‐standardized rates (ASR) of PC incidence and mortality in China were 10.2 and 4.6 per 100 000 males in 2020, respectively, compared to 72 and 8.2 per 100 000 in the United States [1]. A notable difference was also observed in the Surveillance, Epidemiology, and End Results Program (SEER) database, where patients were in the same healthcare system, with the lowest ASR of incidence and mortality in Asian men (53.8 and 8.8 per 100 000), the highest in Black men (171.6 and 38.3 per 100 000), and 97.7 and 17.9 per 100 000 in non‐Hispanic Caucasian men between 2012 and 2018, respectively [3]. The PC discrepancies indicated variations in biological characteristics between ethnicities, in addition to socioeconomic and environmental influences [4]. However, few biological determinants of PC disparities have been identified to date owing to the paucity of genomic data from ethnic minorities, especially Asian men [5].

Previous genomic investigations have found that Asian men have reduced mutation rates of *ERG* fusion and *PTEN* loss and greater rates of *CHD1* deletion, *SPOP* mutations, and *FOXA1* mutations than Caucasian men [6, 7, 8]. However, the PC incidence and mortality in Asian men are not only lower than those of Caucasian men but also lower than those of Black men, although Black men have a similar mutation pattern as Asian men [9, 10]. If these identified genomic differences could solely account for PC disparities, similar, but not opposite, epidemiological trends should be observed in populations with Asian and African ancestries. To facilitate the understanding of the biological factors driving PC disparities, a large‐scale genomic study with Asian PC patients is needed to provide new insights into the genomic characteristics of PC in Asian men.

To address this knowledge gap, we enrolled a prospective sequencing cohort of 1016 Chinese men with PC, including 315 locoregional PC, 313 metastatic castration‐sensitive PC (mCSPC), and 388 metastatic castration‐resistant PC (mCRPC) [11, 12, 13]. We dissected the genomic mutations associated with different ethnicities in 41 genes and explored their mutational characteristics and translational value in PC.

## 2 Materials and methods

### 2.1 FUSCC‐PC cohort

Patients with histologically diagnosed prostate adenocarcinoma and sufficient tissue DNA or ctDNA available for sequencing were enrolled in this study. A total of 1016 PC patients were prospectively recruited at the Fudan University Shanghai Cancer Center (FUSCC) between 08‐05‐2018 and 24‐11‐2021. The genomic testing for patients was ordered by the treating physician at any stage of treatment. We initially planned to utilize a unified ctDNA sequencing platform to profile the somatic aberrations of all patients in this study. However, no mutations were detected from ctDNA for the first 10 patients with localized disease although all patients among them have a high Gleason score (≥ 8) and deep sequencing coverage was achieved [median: 4075X; range: 2166–6067X (Table S1)]. Their archival tumor tissue was retrieved and resequenced to identify whether there were true‐negative mutations. Using the same panels with paired ctDNA, 26 mutations from 10 patients were identified in the tissue DNA samples (Table S2). We thought ctDNA release is insufficient to be captured for men with localized PC and our hypothesis was confirmed in the subsequent research [14, 15]. Finally, for patients with locoregional disease in this study, a tumor tissue DNA sequencing approach was applied. For patients with metastatic disease, ctDNA sequencing was still used due to its real‐time reflection of tumor genomics and inaccessibility of metastatic lesions [16]. Matched tumor biopsy tissues were obtained from 30 men with mPC at the same time to validate the concordance of ctDNA and tissue DNA. ctDNA showed a high sensitivity of 91.2% in detecting mutations present in the paired tumor tissues (Fig. S1, Table S3). Moreover, we performed duplicate genomic testing for 27 patients using a 63‐gene panel, a 128‐gene panel, and a 1460‐gene panel, to confirm the ctDNA fraction and aberrations. All mutations in the overlapped regions between ctDNA panels were validated by each other, and the ctDNA fraction was highly consistent (*R* = 0.99, 0.91, 0.91 for 1460‐gene panel vs. 128‐gene panel, 1460‐gene panel vs. 63‐gene panel, and 128‐gene panel vs. 63‐gene panel, respectively, Fig. S1). A total of 315 patients with locoregional disease undergoing tissue DNA testing, 313 patients with mCSPC, and 388 patients with mCRPC undergoing ctDNA testing were included. Germline mutations in 339 patients in this cohort have been reported previously [17].

### 2.2 Sample processing, DNA isolation, and quantification

Genomic DNA was isolated from formalin‐fixed paraffin‐embedded (FFPE) tumor samples after pathologically verification to select tumor‐rich sections. Whole blood was collected from patients at any stage of their treatment and processed within 72 h after collection in Streck Cell‐Free DNA BCT® tubes at room temperature. To separate plasma and buffy coat, whole blood was centrifuged at 1600 ***g*** for 10 min, and the supernatant was spun by 16 000 ***g*** for an additional 10 min. Buffy coat and liquid biopsy samples were stored at −80 °C until DNA extraction.

Cell‐free DNA (cfDNA) was extracted from plasma using QIAGEN Circulating Nucleic Acids Kit (Qiagen, Hilden, Germany). Germline DNA (gDNA), and tumor tissue DNA were extracted using the DNeasy Blood & Tissue Kit (Qiagen) according to the manufacturer's protocol. DNA concentration was quantified using a Qubit fluorometer 3.0 and Qubit dsDNA High Sensitivity (HS) Assay Kit (Invitrogen, Carlsbad, CA, USA). The DNA fragment length was measured using an Agilent 2100 Bioanalyzer and DNA HS Kit (Agilent Technologies, Santa Clara, CA, USA). The sample quality has to fulfill the criteria as follows: total amount ≥ 20 ng for cfDNA samples with ~ 170 bp fragments, 100 ng for tissue DNA and gDNA with > 1000 bp fragments. The sample processing, DNA isolation, and quantification methods have also been described in our earlier paper [18].

### 2.3 Library construction, sequencing, and quality control

For gDNA and tissue DNA, 100 ng of DNA were sheared with a Covaris E210 system (Covaris) to get ~ 200 bp fragments. All DNA samples underwent library preparation using Accel‐NGS 2S DNA Library Kit (Swift Biosciences, Ann Arbor, Michigan, United States) and xGen Lockdown Probes kit (IDT). The custom xGen Lockdown probe was synthesized by IDT, Inc. for the exons and selected intronic regions of 63, 128, 164, 618, and 1460 genes, respectively. The panels were designed for the detection of mutations and small insertions and deletions. The prepared library was quantified using the Qubit 3.0 Fluorometer, and quality and fragment size were further measured using Agilent 2100 Bioanalyzer (reference fragment size: 280–350 bp; DNA quality: 0.5–50 ng·μL^−1^). Samples underwent paired‐end sequencing on an Illumina Novaseq 6000 platform (Illumina) with 2 × 150‐bp read length. Median coverage of 2593× (range: 201–10 489), 4241× (range: 1523–9762), and 258 (range: 61–2621) was achieved for tumor tissue DNA, plasma cfDNA, and gDNA, respectively. The library construction, sequencing, and quality control methods have also been described in our earlier paper [18].

### 2.4 Data processing and quality control

Raw sequencing data were aligned to the reference human genome (hg19) through Burrows–Wheeler Aligner. After the duplicate removal and local realignment, the Genome Analysis Toolkit and LoFreq were utilized for single nucleotide variation, and short insertions/deletions calling. Variants were then annotated using the annovar software tool [19]. The data processing and quality control methods have also been described in our earlier paper [18].

### 2.5 Germline variants analysis and annotation

Candidate variants identified in gDNA were determined as the valid germline variants for further analysis if they met the following criteria: (a) The allele frequency (AF) was beyond 30%; (b) supporting reads of the allele and variant were at least 15 and 8, respectively; (c) the frequency of the variants was below 1% in the public single‐nucleotide polymorphism databases, including 1000 genomes (https://www.1000genomes.org/), ESP6500 (https://evs.gs.washington.edu/), ExAC (https://gnomad.broadinstitute.org/), and gnomAD (https://gnomad.broadinstitute.org/); (d) the variants were not synonymous SNV; (e) the variants were in the exon or splicing site; and (f) the variants were not present in the inhouse repeat sequence database based on >10 000 cancer patients and healthy men. Germline variants were manually confirmed and annotated as pathogenic, likely pathogenic, of uncertain significance, likely benign, or benign by a molecular pathologist, according to the 2015 American College of Medical Genetics and Genomics and Association for Molecular Pathology consensus criteria [20]. The flow chart of variant annotation has been described previously [17, 21]. Only germline variants classified as pathogenic or likely pathogenic were further analyzed in this study. The germline variants analysis and annotation methods have also been described in our earlier paper [18].

### 2.6 Somatic variants analysis and pathogenicity assessment

The variants identified in ctDNA or tumor tissue sample were defined as the valid somatic variants if they met the following standard: (a) AF was at least 1%; (b) AF was three times higher than the AF of the same variant identified in the matched gDNA; (c) supporting reads of the allele and variant for ctDNA were at least 500 and 10, respectively; (d) supporting reads of the allele and variant for tumor DNA were at least 150 and 5, respectively; (e) the number of forward or reverse strands supporting the allele alteration was at least 5; (f) strand bias at this position was below 60; (g) the frequency of the variant was below 1% in the public germline variants datasets, including 1000 genomes, ESP6500, ExAC and gnomAD; (h) the variant was not synonymous SNV and was in the exon or splicing site; and (i) the variant was not present in the inhouse clonal hematopoiesis variants database.

Pathogenic somatic mutations were identified by the following process: (a) Variants reported as oncogenic or likely oncogenic in OncoKB (http://oncokb.org) were retained and neutral variants were filtered [22]; (b) variants of uncertain significance were further called with ClinVar (https://www.ncbi.nlm.nih.gov/clinvar/; 20210821 version). Variants characterized as pathogenic or likely pathogenic were retained and variants characterized as benign or likely benign were removed. (c) Remaining variants with uncertain significance were further defined with COSMIC annotations (https://cancer.sanger.ac.uk/cosmic). The variant defined as a recurrent mutation according to version 94 of COSMIC database (≥ 3 samples with a missense substitution in the same codon, ≥ 3 samples with an inframe indel in the same codon or > 10 samples with a mutation causing premature protein termination) was considered pathogenic. All other variants were classified as variants of uncertain significance. The analysis herein only included mutations screened as pathogenic. The somatic variants analysis and pathogenicity assessment methods have also been described in our earlier paper [18].

In this study, we focused on 41 genes whose exonic regions were covered by all the sequencing panels (Table S4). The 41 genes were divided into eight pathways based on previous studies [11, 12, 13]. Only variants classified as pathogenic or likely pathogenic were further analyzed in this study.

### 2.7 ctDNA fraction calculation

ctDNA fraction was calculated based on the allele fraction of autosomal somatic mutations. Variants in the genes with copy number amplification were excluded. Mutant allele frequency (MAF) was calculated according to a correction model [23]. ctDNA fraction was estimated as 2/([1/MAF] + 1) due to MAF = (ctDNA*1)/([1 − ctDNA]*2 + ctDNA*1) [23].

### 2.8 Estimation of mutation clonality

The cancer fraction was measured as MAF relative to the ctDNA fraction of this sample based on the assumption that mutation with the maximum allele frequency is present in every cancer cell. Mutations with cancer fraction > 25% were considered as clonal whereas mutations with cancer fraction < 25% were categorized as subclonal [24].

### 2.9 Comparison of FUSCC‐PC dataset with TCGA/MSKCC/SU2C datasets

The genomic variants, clinical and ethnic data were accessed from cBioPortal (https://www.cbioportal.org/) for the TCGA cohort (Firehose Legacy) [25], MSKCC cohort (MSK, Clin Cancer Res 2020) [26] and SU2C cohort (SU2C/PCF Dream Team, Cell 2015) [27]. The ethnic data downloaded from cBioPortal only included 154 patients with available race categories. Thus, more complete self‐reported ethnicity data for patients in the TCGA cohort was further obtained from Supplementary table 2 from the publication by Jiao Yuan et al [10]. We excluded 84 patients from non‐Caucasian races and unknown ancestry in the TCGA cohort to ensure a high‐quality comparison of genomic mutations between East Asian men and Caucasian men. Finally, 408 Caucasian patients in TCGA cohort were included in our analysis. In the MSKCC cohort, 312 patients self‐reported as Caucasian ethnicity were retained and 112 non‐Caucasian patients were excluded for subsequent analysis. According to the clinical annotations, patients from TCGA cohort, MSKCC cohort, and SU2C cohort represented the locoregional PC men, mCSPC men, and mCRPC men, respectively.

To account for differences in sequencing depth across cohorts, mutations with allele frequency < 1% were removed. Moreover, somatic mutations in 41 genes from three datasets were annotated using the same bioinformatics pipeline as the FUSCC‐PC dataset described above.

### 2.10 Comparison of FUSCC‐PC cohort with other two East Asian PC cohorts

We utilized publicly available somatic genomic calls from East Asian PC cohort to explore whether FUSCC‐PC cohort is representative of a larger East Asian PC population. The genomic profiles and clinical characteristics of 292 patients in Renji (Chinese) cohort were retrieved from Supplementary tables S3 and S5 from their publication [28]. A total of 24 genes overlapped between FUSCC‐PC cohort and Renji cohort were included in the comparative analysis. For the Japanese cohort, detailed mutation calls were taken from Supplementary table S4, and there were 33 genes covered in both cohorts [29]. All patients in both cohorts were in mCRPC stage and received ctDNA sequencing. The mutation annotation methodology applied in both cohorts was consistent with the FUSCC‐PC cohort.

### 2.11 Pan‐prostate cancer *FOXA1* mutation analysis

Mutation calls of 7818 samples from 8050 patients from 20 studies were downloaded from cBioPortal. Duplicated samples were pruned and only patients with identified Caucasian ancestry and available sample organ data were retained to generate a Western cohort comprising 2330 patients (1698 primary PC and 632 metastasis). FUSCC‐PC cohort (*n* = 859), Chinese Prostate Cancer Genome and Epigenome Atlas (CPGEA) cohort (*n* = 208), Renji cohort (*n* = 292), and Japanese cohort (*n* = 100) were combined into an Eastern Asian cohort (*n* = 1459, 523 primary PC and 936 metastasis) [7, 28, 29]. The genomic data from Renji cohort and Japanese cohort were downloaded as described earlier, and genomic data in the CPGEA cohort were obtained from Supplementary data 8 in the publication [7]. The *FOXA1* mutations were classified into class‐1 and class‐2 based on their position relative to amino acid residue 275, according to the previous study [30]. Mutations between H247 and E269 were defined as Wing2 variants.

### 2.12 Statistical analysis

Comparisons between groups were performed using Fisher's exact test for categorical variables and the Mann–Whitney *U* test for continuous variables. Differences in mutation frequency between ethnicities for each phenotype (locoregional PC, mCSPC, and mCRPC) were further tested using a multivariable logistic regression model adjusted for clinical features. Cox proportional hazards regression analysis was used to evaluate the association between genomic alterations and clinical outcomes. All the reported *P*‐values were two‐tailed. Multiple testing correction was applied using the false discovery rate (*q*‐value) method, and *q* < 0.05 was considered statistically significant. All analyses were performed using r version 4.1.0 (www.R‐project.org).

### 2.13 Ethics approval and consent to participate

This study was approved by the ethics committee at FUSCC (Ethical code: 050432‐4‐1911D) and conducted in accordance with the Declaration of Helsinki. Written informed consent was obtained before enrollment.

## 3 Results

### 3.1 Mutational landscapes across disease stages in Chinese prostate cancer patients

**Fig. 1:**
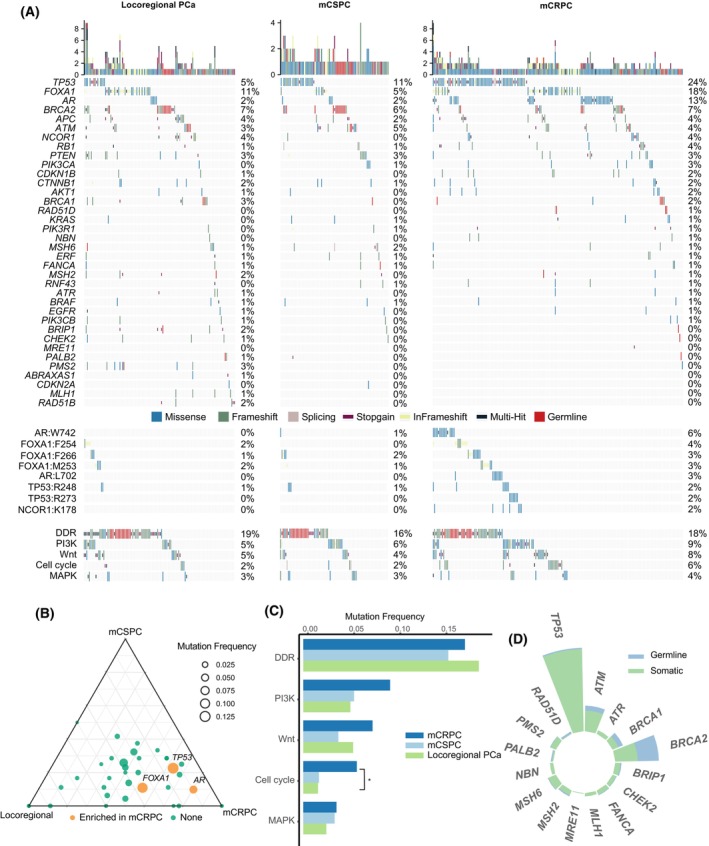
Genomic mutations across disease stages in FUSCC‐PC cohort. (A) Mutational profile of 41 selected genes in 859 patients in terms of gene, variant, and pathway levels. The mutations were SNVs and Indels likely to be pathogenic and were displayed by gene for each patient. The numbers on the right side were the mutation counts in each gene and each stage. Mutation frequency was defined as the ratio of the number of patients with mutations relative to the total number of patients at a given clinical stage. Hotspot variants (mutation frequency > 2% in mCRPC) were shown to indicate the castration‐resistance associated mutations. (B) Enrichment of prevalence of mutated genes in tumors across disease stages. Comparisons were corrected by the false discovery rate. Each point corresponds to a gene. Its position represents its relative abundance with respect to each disease stages, and its size represents the average frequencies across all three stages. Orange circles represent genes enriched in one stage compared with the others, whereas green circles represent genes that are not significantly enriched in a specific disease stage. (C) Enrichment of mutation prevalence in pathways across disease stages. Comparisons were corrected by the false discovery rate. (D) The proportion of germline mutations and somatic mutations in 16 DDR genes and *TP53*, respectively. DDR, DNA damage repair; mCRPC, metastatic castration‐resistant prostate cancer; mCSPC, metastatic castration‐sensitive prostate cancer; SNV, single nucleotide variant.

This study enrolled 1016 patients (Fig. S2, Table S5). In 77.6% (544/701) of the metastatic PC patients, the ctDNA fraction was greater than the detection threshold (2%). To reduce false‐negative results attributed to low tumor content, we limited our final analysis to 859 patients (315 locoregional PC, 236 mCSPC, and 308 mCRPC) with quantifiable ctDNA or tissue DNA sequencing (Fig. 1A, Fig. S3). A total of 700 pathogenic and likely pathogenic mutations were identified in the 859 patients (Table S6). In locoregional PC, the most commonly mutated genes were *FOXA1* (11.4%), *BRCA2* (7%), *TP53* (5.1%), *APC* (3.8%), *NCOR1* (3.5%), *ATM* (3.2%), and *PTEN* (3.2%). In mCSPC, the most frequently mutated genes were *TP53* (11%), *BRCA2* (6.4%), *ATM* (4.7%), *FOXA1* (4.7%), and *PTEN* (2.5%). The most prevalent mutations observed in mCRPC were *TP53* (23.7%), *FOXA1* (17.5%), *AR* (13.3%), *BRCA2* (6.8%), *APC* (4.2%), and *ATM* (4.2%) (Fig. 1A, Fig. S4). A comparison of mutation frequencies among different disease stages revealed that *AR* and *TP53* were enriched in mCRPC compared with locoregional PC (13% vs. 1.6%, *q* < 0.001; 23.7% vs. 5.1%, *q* < 0.001) or mCSPC (13% vs. 2.1%, *q* < 0.001; 23.7% vs. 11%, *q* = 0.0015; Fig. 1B). *FOXA1* mutations were more frequently observed in mCRPC than in mCSPC (17.5% vs. 4.7%, *q* < 0.001; Fig. 1B). In addition, mutations in the cell cycle pathway occurred at a higher rate in mCRPC than in locoregional PC (5.8% vs. 1.6%, *q* = 0.026, Fig. 1C).

Because of their potential susceptibility functions, we investigated the proportion of germline and somatic mutations in the 16 DNA damage repair (DDR) genes and *TP53*. In the 16 DDR genes, germline mutations accounted for a low proportion of total mutations (germline: 6.3% vs. somatic: 15.7%, Fig. 1D), especially in *RAD51D* (0.3% vs. 0.1%), *PALB2* (0.2% vs. 0.2%), *BRCA2* (3.4% vs. 3.6%), and *BRCA1* (0.8% vs. 1%). However, rare germline events relative to somatic mutations were observed in *TP53* (germline: 0.2% vs. somatic: 13.2%, Fig. 1D). Notably, a recent study reported that inherited *TP53* mutations elevated the risk of PC and occurred in approximately 0.6% of patients in the Western cohort [31]. Only 0.2% of patients harbored inherited pathogenic mutations in *TP53* in our current study, however, and no *TP53* pathogenic mutations were detected in our previous multi‐institutional cohort [17].

### 3.2 Clinical and pathological genomic associations in prostate cancer

**Fig. 2:**
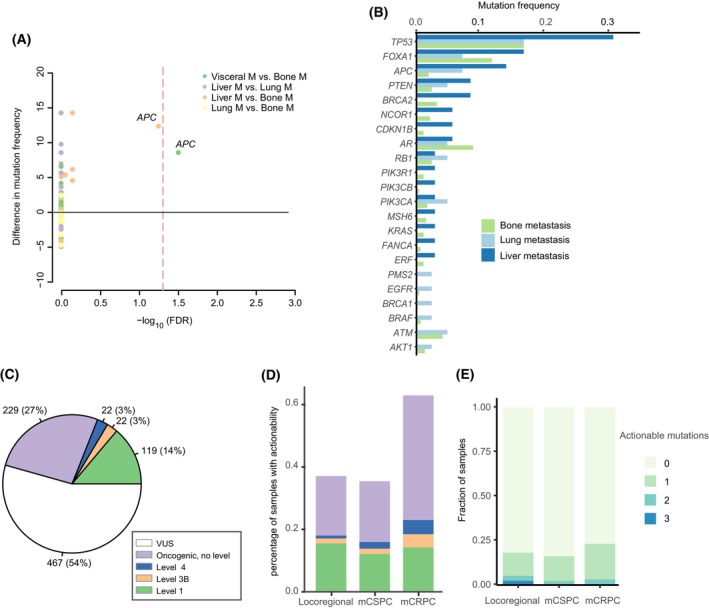
Clinical and pathological genomic associations. (A) The associations between oncogenic genomic mutations and organ‐specific metastatic patterns. FDR is the adjusted *P* value using the Benjamini–Hochberg procedure. (B) Mutation frequency in selected genes split by metastatic organs in FUSCC‐PC cohort. (C) Distribution of samples with different actionable levels according to the OncoKB database. Only the highest‐level actionable mutation is represented per patient. (D) Frequencies of samples with actionability across disease stages. (E) Fractions of samples with multiple actionable mutations in different clinical stages. FDR, false discovery rate; M, metastasis; mCRPC, metastatic castration‐resistant prostate cancer; mCSPC, metastatic castration‐sensitive prostate cancer; VUS, variant of uncertain significance.

We analyzed the genomic makeup of metastases (*N* = 544) according to organ location and found *APC* mutations were significantly more frequent in visceral metastases than in bone metastases (10.5% vs. 1.9%, *q* = 0.03, Fig. 2A,B). We further explored potentially actionable targets in Chinese patients with prostate cancer according to OncoKB annotations (Fig. S5). A similar prevalence of Level 1 actionable biomarkers was observed across disease stages, comprising 14% of Chinese PC patients (Fig. 2C,D). Interestingly, 27 patients from our cohort harbored more than one actionable mutation, with multiple mutations in the DDR pathway being the dominant comutation pattern (Table S7), indicating the potential for extraordinary benefits from poly (ADP‐ribose) polymerase (PARP) inhibitors for these patients. To investigate the clonality of individual variants, we estimated the cancer fraction of mutations in the top mutated genes in metastatic PC. Mutant alleles within the *BRCA1*, *BRCA2*, *MSH6*, *ATM*, and *FANCA* genes were detected at clonal frequencies (Fig. S6A,B). However, five (5/21, 24%) variants in *ATM*, four (4/19, 21%) in *BRCA2*, two (2/4, 50%) in *FANCA*, and one (1/8, 12.5%) in *MSH6* demonstrated subclonal status. Furthermore, all mutations detected in *MSH2*, *RAD51D*, and *NBN* in our cohort were sub‐clonal.

### 3.3 Genomic differences between Chinese and Caucasian men

**Fig. 3:**
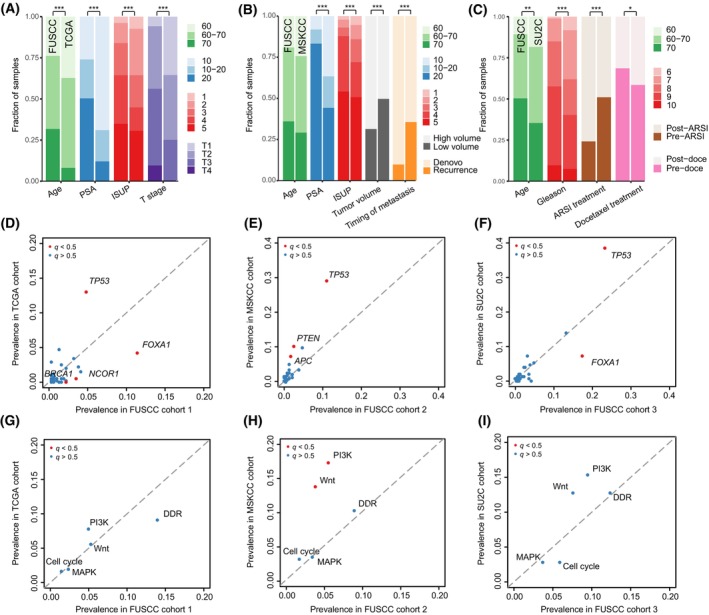
Comparison of FUSCC‐PC dataset with TCGA/MSKCC/SU2C datasets. (A–C) Clinical features of 3 cohorts in FUSCC‐PC dataset compared with three publicly available sequencing datasets from the PCa with Caucasian men (FUSCC‐cohort 1 vs. TCGA cohort; FUSCC‐cohort 2 vs. MSKCC cohort; FUSCC‐cohort 3 vs. SU2C cohort). Comparisons between groups were performed using Fisher's exact test for categorical variables and the Mann–Whitney *U* test for continuous variables. “*” indicates *P* < 0.05, “**” indicates *P* < 0.01, “***” indicates *P* < 0.001. (D–F) Scatter plots of the prevalence of mutated genes from the FUSCC‐PC and the Caucasian cohorts. (G–I) Scatter plots of the mutation frequency in pathways from the FUSCC‐PC and the Caucasian cohorts. ARSI, androgen receptor signaling inhibitors; ISUP, International Society of Urological Pathology; PSA, prostate specific antigen.

We systematically reviewed previous transancestry clinicopathological studies using a unified sequencing platform and evaluated the proportion of Asian patients with PC enrolled in these cohorts. Almost all studies underrepresented Asian men (Fig. S7) and did not have enough statistical power to detect certain differences in genomic mutations, despite recognized discrepancies between ethnicities [26, 32, 33, 34, 35]. We focused on pathogenic variants and adjusted for clinical features associated with tumor genomics due to the significantly different distribution of patient characteristics across ethnicities, as shown in Fig. 3A–C (Table S8).

Compared with the TCGA cohort, three genes (*FOXA1*, *q* = 0.004; *NCOR1*, *q* = 0.028; *BRCA1*, *q* = 0.028, Fig. 3D) were more prevalent in our cohort, and one gene (*TP53*, *q* = 0.004, Fig. 3D) was mutated at a lower rate in our dataset. Notable differences in *FOXA1* [11.4% vs. 4.2%, adjusted HR = 3.41, 95% confidence interval (CI): 1.69–6.86; *q* = 0.01, Table S9] and *TP53* (4.8% vs. 13%, adjusted HR = 0.25, 95% CI: 0.13–0.5; *q* = 0.002, Table S9) persisted even after adjusting for clinical factors [age, T stage, and International Society of Urological Pathology (ISUP) grade]. In the mCSPC group, enrichment for mutations in *TP53* (11% vs. 29.2%, *q* < 0.001, Fig. 3E), *PTEN* (2.5% vs. 10.3%, *q* = 0.005, Fig. 3E), and *APC* (1.7% vs. 7.4%, *q* = 0.024, Fig. 3E) were identified in the MSKCC cohort. Multivariable logistic regression correcting for age, timing of metastasis, tumor volume, and ISUP grade further proved this association to be robust (*TP53*, adjusted HR: 0.3, 95% CI: 0.18–0.49, *q* < 0.001; *PTEN*, adjusted HR = 0.19, 95% CI: 0.08–0.48, *q* = 0.006; *APC*, adjusted HR = 0.15, 95% CI: 0.05–0.48, *q* = 0.01, Table S10). In line with the differences in gene‐level mutations, PI3K (5.5% vs. 17.3%, adjusted HR = 0.26, 95% CI: 0.13–0.5, *q* < 0.001) and Wnt (3.8% vs. 13.8%, adjusted HR = 0.22, 95% CI: 0.1–0.49, *q* < 0.001, Fig. 3H, Table S10) pathway mutations were more common in Western mCSPC patients. Unexpectedly, in mCRPC, the mutational profile was broadly similar between the FUSCC‐PC and SU2C cohorts. Although differences were observed in *FOXA1* (17.5% vs. 7.3%, Fig. 3F) and *TP53* (23.4% vs. 38.7%, Fig. 3F), these were mainly driven by disease extent instead of ethnic differences (*FOXA1*: *q* = 0.06; *TP53*: *q* = 0.06, Table S11). Collectively, the genomic differences between ethnicities were mainly enriched in the locoregional PC and mCSPC groups (Fig. S8).

We also compared our dataset with publicly available genomic data from two other East Asian cohorts: the Renji (Chinese) cohort (*n* = 292) and the Japanese cohort (*n* = 100). All genes covered in both cohorts demonstrated a similar mutation rate between the FUSCC‐PC cohort and the Renji cohort (Fig. S9) or between the FUSCC‐PC cohort and the Japanese cohort (Fig. S10), highlighting common findings of genomic mutations in three independent ethnically matched cohorts.

### 3.4 Clinical implications of genomic features in Chinese prostate cancer patients

**Fig. 4:**
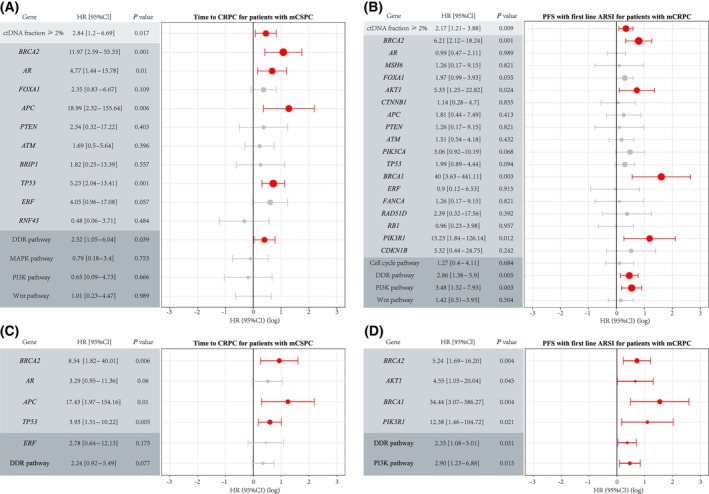
Clinical implications of genomic features in Chinese prostate cancer patients. (A) Univariate Cox proportional hazards regression results of time to CRPC in mCSPC patients (*n* = 143). (B) Univariate Cox proportional hazards regression results of progression‐free survival with first‐line ARSI in mCRPC patients (*n* = 82). (C) Adjusted Cox proportional hazards regression results of time to CRPC in mCSPC patients (*n* = 143). Note: adjusted for ctDNA fraction, age at test and treatment types. (D) Adjusted Cox proportional hazards regression results of progression‐free survival with first‐line ARSI in mCRPC patients (*n* = 82). Adjusted for ctDNA fraction and age at test. Cox proportional hazards regression analysis was used to evaluate the association between genomic alterations and clinical outcomes. PFS, progression‐free survival.

We further explored the prognostic role of genomic mutations, particularly mutations in genes with diverse mutation rates between ethnicities (i.e., *BRCA2*, *TP53*, *APC*, and *FOXA1*). We restricted our analysis to a subset of patients with mCSPC (*n* = 143) and mCRPC who received androgen receptor signaling inhibitors (ARSI) as first‐line therapy for mCRPC (*n* = 82). All 225 patients underwent ctDNA testing before or within 1 week of therapy initiation (Fig. S11). Mutations in *BRCA2*, *TP53*, and *APC* were associated with a higher risk of developing mCRPC, and this relationship remained significant after correcting for ctDNA fraction, age, and treatment types in our mCSPC cohort (Fig. 4A,C, Table S12). *BRCA2* mutations were also associated with a shorter time of treatment with a first‐line ARSI, and *TP53* mutations indicated unfavorable overall survival with first‐line ARSI treatment, although there was no association between *APC* mutations and survival on ARSI (Fig. 4B,D, Fig. S12, Table S13). Mutations in *FOXA1* did not show a significant association with either time on therapy with androgen deprivation therapy (ADT) or ARSI or with overall survival. In addition, ctDNA fractions > 2% showed robust discrimination for a shorter time to mCRPC and poor survival with ARSI (Fig. 4A,B, Fig. S12, Tables S12 and 13).

### 3.5 Comprehensive comparative analysis of *FOXA1* mutations between East Asian and Caucasian men

**Fig. 5:**
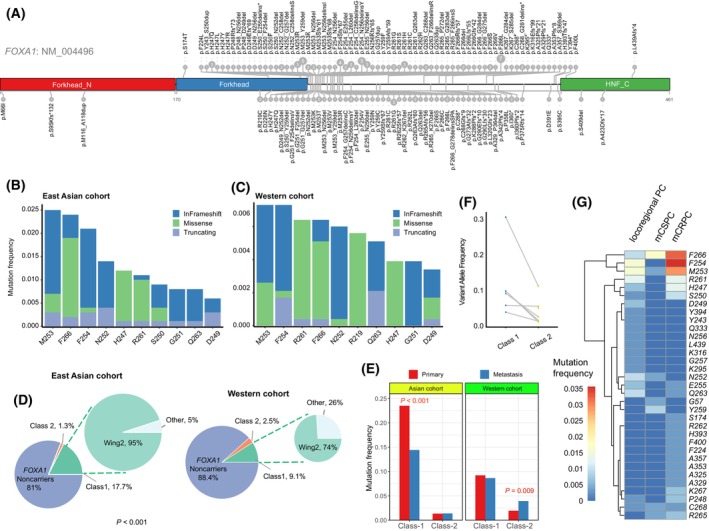
Comparison of mutation spectrum in *FOXA1* between tumors with East Asian and Caucasian men. (A) Distribution plot of *FOXA1* mutations in FUSCC‐PC cohort (top) and TCGA/MSKCC/SU2C cohorts (bottom). (B) The top 10 mutated variants in *FOXA1* in the East Asian cohort (*n* = 1459). (C) The top 10 mutated variants in *FOXA1* in the Caucasian cohort (*n* = 2330). (D) Distribution of *FOXA1* mutations observed in East Asian cohort and Caucasian cohort. Mutations were classified into several classes based on their location in the *FOXA1* protein. (E) Frequency of *FOXA1* mutational classes by prostate cancer stages and races (*n* = 523 primary PC and 936 metastases in Asian cohort; *n* = 1698 primary PC and 632 metastases in Western cohort). (F) Variant allele frequency of six paired *FOXA1* class‐1 and class‐2 mutations in East Asian cohort. (G) The heatmap showing the frequency of *FOXA1* variants across disease stages in FUSCC cohort. Comparisons of *FOXA1* mutations between groups were performed using Fisher's exact test.

Due to the obvious difference in *FOXA1* between tumors from East Asian and Caucasian patients, supported by evidence from multiple studies [7, 25, 34, 35, 36, 37], we performed a comprehensive comparative analysis of *FOXA1* mutants between ethnicities. We first explored the mutational spectrum of *FOXA1* in our cohort and revealed trends similar to those of a previous study [7], with a high enrichment of *FOXA1* hotspot variants in Chinese men with PC (Fig. 5A). We further synthesized our cohort with previously reported cohorts for an East Asian cohort, including 1459 patients (523 locoregional PC and 936 mPC) [7, 28, 29]. An aggregate cohort comprising 2330 Caucasian patients (1698 primary PC and 632 mPC) was also developed using public data (Table S14) [13, 25, 26, 34, 37, 38, 39, 40, 41, 42, 43, 44, 45, 46, 47, 48, 49, 50, 51]. Next, we investigated whether the top mutated variants differed between tumors from Asian and Caucasian men. The top 10 mutated variants in the East Asian cohort were M253 (2.5%), F266 (2.3%), F254 (2.1%), N252 (1.4%), H247 (1.2%), R261 (1.1%), S250 (0.8%), G251 (0.8%), Q263 (0.8%), and D249 (0.6%) (Fig. 5B), which was highly concordant with the top mutated variants in the Western cohort, despite occurring at a lower rate (Fig. 5C). According to prior research, *FOXA1* mutations were classified into class‐1 and class‐2 based on their position relative to amino acid residue 275 (class‐1 is before 275, class‐2 is after 275). Class‐1 mutations originate in early prostate cancer, whereas class‐2 mutations are acquired in metastatic prostate cancers [30]. Because of the varying roles different *FOXA1* structural classes play in oncogenesis, we further tested whether there was a difference in class‐1 and class‐2 mutation frequencies between cohorts. A higher mutation rate of class‐1 (17.7% vs. 9.1%, *P* < 0.001) was found in tumors in East Asian men. Surprisingly, class‐2 mutations showed the opposite trend, with the East Asian cohort having fewer class‐2 mutations (1.3% vs. 2.5%, *P* = 0.018; Fig. 5D). Furthermore, Class‐2 mutated at a higher prevalence in metastasis than primary PC in Western men, which is consistent with the previous studies [30]. However, this was not observed in Asian men. In addition, we found a lower prevalence of class‐1 mutations in metastasis compared with primary PC in Asian men (Fig. 5E, Table S15). We also evaluated the clonal status of class‐1 and class‐2, using six paired variants detected from ctDNA. The variant allele frequency (VAF) of class‐2 mutations was significantly lower than that of class‐1 mutations in the same patient (Fig. 5F), suggesting that class‐2 mutations were more likely to be subclonal, a finding that is consistent with a previous study [30]. Then, we sought to uncover the reason why the rate of *FOXA1* mutations was higher in mCRPC compared with mCSPC and Locoregional PC. We hypothesized there existed heterogeneity within class‐1 variants, considering the increase in *FOXA1* mutation frequencies is mostly due to class‐1 mutations (Table S15). We found some variants enriched in mCRPC, such as F266 and F254 (Fig. 5G), which were reported to promote tumor growth in a seminal study [52]. This finding, to a certain extent, confirmed our above hypothesis.

## 4 Discussion

To the best of our knowledge, we established the largest clinicogenomic dataset of Chinese PC by including patients from all three disease stages with robust clinical information and a unified analysis pipeline. The largest previous study on Chinese PC comprised 306 tumor samples [28]. Our work serves as a valuable resource, greatly increasing the amount of sequencing data and furthering our understanding of the molecular basis of PC in China.

As a combination of several metastases from one individual [53], ctDNA may reflect different genomic mutations compared to those detected in only one tumor tissue. However, the same research group also reported that a ctDNA‐based assay is sufficient to identify all driver DNA alterations that are present in matched tissue [16]. Our data indicated that our ctDNA‐based platform could capture 91.2% (52/57) of the variants present in the paired tissues (Fig. S1), which is consistent with the previous study (93.6%) [16], although some differences exist between ctDNA and tissue. This supports the subsequent comparative analysis between the FUSCC cohort (ctDNA sample) and the MSKCC (tissue sample) or the SU2C cohort (tissue sample).

Our analysis comparing genomic mutations between clinical stages revealed that mutations in *TP53*, *AR*, *FOXA1*, and cell cycle pathways were enriched in mCRPC patients. These results are consistent with those of previous studies [13, 27, 51, 54], reiterating the association between these genomic mutations and castration resistance in a cohort with different genetic backgrounds. We also compared the mutation prevalence according to metastatic sites and found that patients with visceral metastasis, especially those with liver metastasis, had a higher frequency of *APC* mutations, which is in line with the findings from the MSK‐MET cohort that studied 1762 PC patients [55]. It is worth noting that *APC* is also a frequently mutated gene in digestive system tumors [56]. How *APC* mutations cause PC cells to acquire dual organotropism in the liver and lungs is an interesting question that warrants further experimental investigation.

Our findings on the genomic characteristics of PC in Chinese men from multiple aspects, including mutation enrichment, variant allele frequency, and metastatic pattern drivers, were generally consistent with those of previous studies [13, 56, 57, 58]. However, our study did not simply validate the genomic characteristics of PC. Instead, the importance of our work was the comprehensive analysis of genomic mutations in 41 genes in tumors from both Chinese and Caucasian men.

For the first time, we noted that genomic differences across ethnicities mainly occurred in locoregional PC and mCSPC, whereas mCRPC demonstrated a similar genomic spectrum. This phenomenon may suggest that PC in Chinese men harbors different molecular bases at an early stage compared with tumors in Caucasian men due to diverse oncogenic drivers between ethnicities. These may include environmental exposure, germline genetics, and lifestyle factors, but it seems the tumors acquired similar mutations under selective pressure from drugs during their development into mCRPC. Moreover, we found that patients carrying mutations in genes with varying mutation rates between ethnicities (e.g., *TP53*, *PTEN*, and *APC*) tended to have unfavorable outcomes. A series of studies based on SEER and other population‐based registries reported that Asian men have better prognoses than Caucasian men in localized and metastatic castration‐sensitive settings [59, 60], whereas no survival difference between ethnicities was observed in the mCRPC stage in a pooled analysis that included nine phase III clinical trials in which patients had equal access to care [61]. Our findings provide biological evidence partially accounting for the observed survival disparities at different clinical stages.

More importantly, our data revealed that the molecular basis of PC should be incorporated into the treatment decisions of patients in the CSPC stage from different ethnicities, whereas mCRPC patients from both ancestries can benefit equally if they have equal access to precision treatment. The CHART trial recently reported positive results, comparing the efficacy of SHR3680 and bicalutamide in combination with ADT in patients with high‐volume mCSPC [62]. The median radiographic progression‐free survival (rPFS) in the bicalutamide group in the CHART study was 23.5 months. However, the median rPFS in the placebo group in the LATITUDE trial (abiraterone combined with ADT vs. ADT in mCSPC) was only 14.8 months, whereas it was 22.1 months in the placebo group in the TITAN trial (apalutamide combined with ADT vs. ADT in mCSPC) [63, 64]. Despite having a higher disease volume than the other two trials, patients in the CHART trial appeared to benefit more from androgen receptor inhibitors. A major difference between trials was that 90.5% of participants in the CHART trial were Chinese patients, whereas the LATITUDE and TITAN trials were mainly Caucasian patients. The lower mutation rate in *TP53*, *PTEN*, and *APC* and the higher mutation rate in *FOXA1* in Chinese prostate cancer patients may be associated with an exceptional response to ARSI.

We systematically explored the differences in the mutation spectrum of *FOXA1* between East Asian and Caucasian men. We found that the top mutated variants in East Asian patients also exhibited a relatively high mutation frequency in the Western cohort. According to the data reported by previous studies, a higher mutation frequency of *FOXA1* has been confirmed in both Chinese and Asian‐American cohorts when compared to Caucasian ones [7, 32, 34, 35, 65], indicating that this distinction mainly results from differences in germline genetics rather than environmental factors. However, just how germline mutation patterns almost exclusively in East Asian patients affect these somatic events in *FOXA1* remains unclear and needs to be explored in future studies. Our study found that patients from East Asia have more *FOXA1* class‐1, which is consistent with previous studies. In addition, there was no enrichment of class‐2 mutations in metastatic tumors relative to primary tumors observed in East Asian men, which is in contrast to the findings in Caucasian patients [30]. Class‐2 mutations would increase DNA affinity and drive metastasis via the Wnt pathway through TLE3 inactivation [30]. Tumors in East Asian men rarely acquire the ability to metastasize through class‐2 mutations. Additionally, the data from the GENIE 7.0 and 8.0 cohorts further validated race‐specific changes in *FOXA1* mutations. In Asian men, *FOXA1* mutated at a lower rate in metastasis than primary PC (~ 40% vs. ~ 15%), whereas in Western men, *FOXA1* mutated at a similar rate across stages [32, 35]. Taken together, we could conclude that the lower rate of *FOXA1* mutations in mCSPC is Asian‐specific and is mostly attributed to the decrease in class‐1 mutation rates. Furthermore, the higher incidence of *FOXA1* mutations in mCRPC compared to mCSPC and locoregional prostate cancer prompted us to investigate the underlying factors contributing to this disparity. Our analysis revealed the presence of certain variants within the class‐1 variants, such as F266 and F254, which have been previously associated with promoting tumor growth based on seminal research [52] may explain the higher incidence of *FOXA1* mutations in mCRPC. These findings highlight the underlying mechanistic differences in driving PC progression through different genetic backgrounds and provide a new paradigm for the interplay between inherited and somatic events in cancer phenotypes.

Although our comparative analysis of well‐matched PC cases from Chinese and Caucasian men in the same clinical setting demonstrated genomic diversity between patients of different ancestries, there were several limitations to our study. First, our analysis focused on a limited number of genes, although these 41 genes were among the most important PC drivers. Additional studies are needed to explore the biological differences driving PC disparities at the whole‐genome or even multi‐omics levels. Furthermore, our research was not a head‐to‐head study that included patients from different ethnicities using a unified sequencing and bioinformatics analysis platform. However, we provided the largest sample cohort of Chinese PC patients with genomic data and controlled for confounding factors that may affect genomic mutations to ensure the reliability of the analysis. Finally, the follow‐up period of the prospective study is short and ongoing, which may provide more clinically relevant information in the near future.

## 5 Conclusions

Our findings not only provide evidence of remarkable molecular variations in CSPC, but also a comparable spectrum of mutations in CRPC between Chinese and Western men. Although mCRPC patients of both ethnicities can benefit equally if they have equal access to precision medicine, the molecular bases underlying the condition should be incorporated into treatment decisions for CSPC patients of different ethnicities. Discovering fundamental differences between ethnicities paves the way for global patient‐centered care while also encouraging inclusivity in genomic‐driven studies.

## Conflict of interest

The authors declare no conflict of interest.

## Author contributions

YZ, DY, and YW designed this study. TZ, YW, JP, WG, XQ, BD, GL, HG, and SJ obtained the data. YW, TZ, BW, JW, and BF analyzed and interpreted the data. YW, TZ, JW, and BW drafted the manuscript. YZ, JW, WG, XQ BD, GL, and HG reviewed the manuscript. YW, TZ, and BW performed statistical analysis. YZ, DY, and JW obtained funding and supervised. WG, XQ, BD, GL, and HG gave administrative, technical, and material support. All authors read and approved the final manuscript.

6

### Peer review

The peer review history for this article is available at https://www.webofscience.com/api/gateway/wos/peer‐review/10.1002/1878‐0261.13511.

## Supporting information


**Fig. S1.** Validation of variants detected by ctDNA sequencing.
**Fig. S2.** Cohort summary and sample distribution.
**Fig. S3.** Mutational profile of 41 selected genes in patients in FUSCC‐PC cohort classified by treatment and annotated with the variation type and mutation frequency.
**Fig. S4.** Mutational profile in localized PC.
**Fig. S5.** Levels of actionable mutations according to the OncoKB database.
**Fig. S6.** Distribution of Variant allele frequency in the genes with mutation frequency ≥1% (A) and split by disease stages (B).
**Fig. S7.** Distribution of participants from different ancestries in the previous genomic studies using a unified sequencing platform.
**Fig. S8.** Mutation frequencies across stages and races.
**Fig. S9.** Comparison of genomic mutations between FUSCC‐PC cohort 3 with Renji cohort.
**Fig. S10.** Comparison of genomic mutations between FUSCC‐PC cohort 3 with Japanese cohort.
**Fig. S11.** Mutational profile of 41 selected genes in a subset of patients classified by treatment and annotated with the variation type and mutation frequency.
**Fig. S12.** Univariate Cox proportional hazards regression results of overall survival with first‐line ARSI in mCRPC patients (n = 82).

**Table S1.** Clinical data of patients with locoregional disease included in ctDNA sequencing analysis (n = 10).
**Table S2.** Somatic variants revealed by tissue DNA sequencing from the 10 locoregional patients receiving ctDNA sequencing.
**Table S3.** High concordance of variants detected between ctDNA and tissue DNA.
**Table S4.** Complete list of Genes of Interest.
**Table S5.** Clinical Characteristics of Patients in FUSCC‐PC cohort.
**Table S6.** Summary of detected pathogenic variants in FUSCC‐PC cohort.
**Table S7.** Patient‐based actionable mutations and corresponding drugs in FUSCC‐PC cohort.
**Table S8.** Comparison of patient characteristics between FUSCC‐PC cohort and TCGA/MSKCC/SU2C cohorts.
**Table S9.** Associations of all genomic mutation frequencies with ISUP, race, T stage, and age using multivariable logistic regression in locoregional patients.
**Table S10.** Associations of genomic mutation frequencies with ISUP, race, age, timing of metastasis, and tumor volume using multivariable logistic regression in mCSPC patients.
**Table S11.** Associations of genomic mutation frequencies with Gleason Score, race, age, and previous therapies using multivariable logistic regression in mCRPC patients.
**Table S12.** Association of genomic mutations with time to mCRPC in a subset of mCSPC patients (n = 143).
**Table S13.** Association of common genomic alteration and ctDNA fraction with overall survival and progression‐free survival with first‐line ARSI in mCRPC patients (n = 82).
**Table S14.** Complete list of FOXA1 mutations in our study.
**Table S15.** Frequency of FOXA1 mutational classes across disease stages in FUSCC cohort.
