# Supplementary material for: Prospective clinical sequencing of 1016 Chinese prostate cancer patients: uncovering genomic characterization and race disparity
Source: Mol Oncol. 2023 Aug 23;17(10):2183–99. doi: 10.1002/1878-0261.13511 (PMC10552897; doi:10.1002/1878-0261.13511)

**Supplementary Figure 1: The validation of variants detected by ctDNA sequencing. A.** Variants detected in ctDNA and matched tumor tissue from 30 patients. **B-D.** Concordance between genomic mutations detected using 63-gene, 128-gene and 1460-gene panels. **E-G.** Concordance between ctDNA fraction detected using 63-gene, 128-gene and 1460-gene panels.


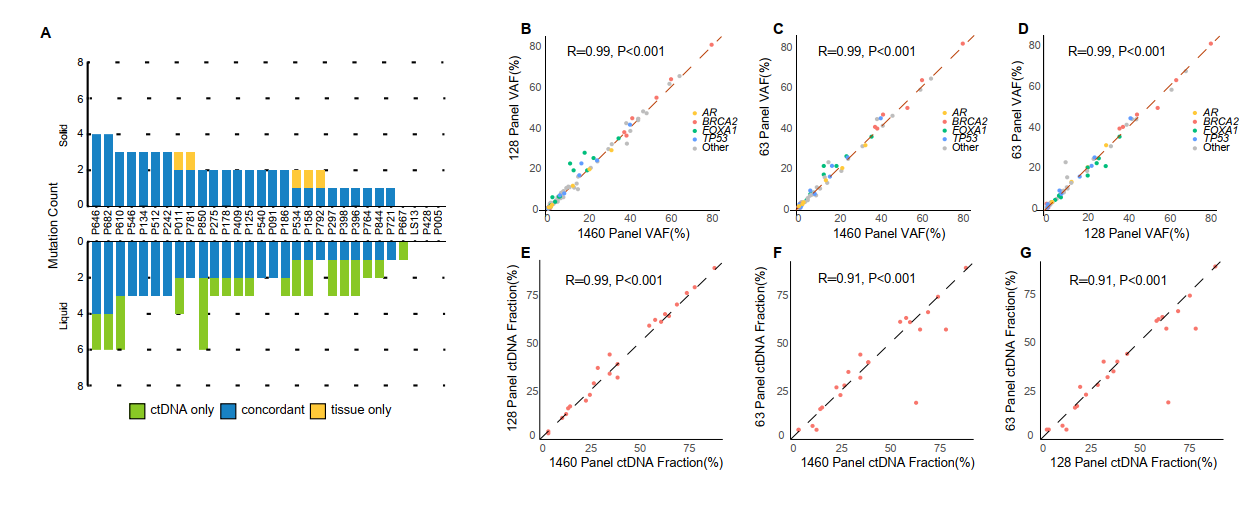


**Supplementary Figure 2. Cohort summary and sample distribution.** Tumor tissue samples and cell**-**free DNA were collected from the 315 locoregional PC, 701 mPC patients, respectively. Patients in 3 cohorts were divided according to clinical stages and were referred to sequencing to identify clinically relevant genomic information and optimize treatment options. PC= prostate cancer; mPC= metastatic prostate cancer; mCSPC= metastatic castration-sensitive prostate cancer; mCRPC= metastatic castration-resistant prostate cancer


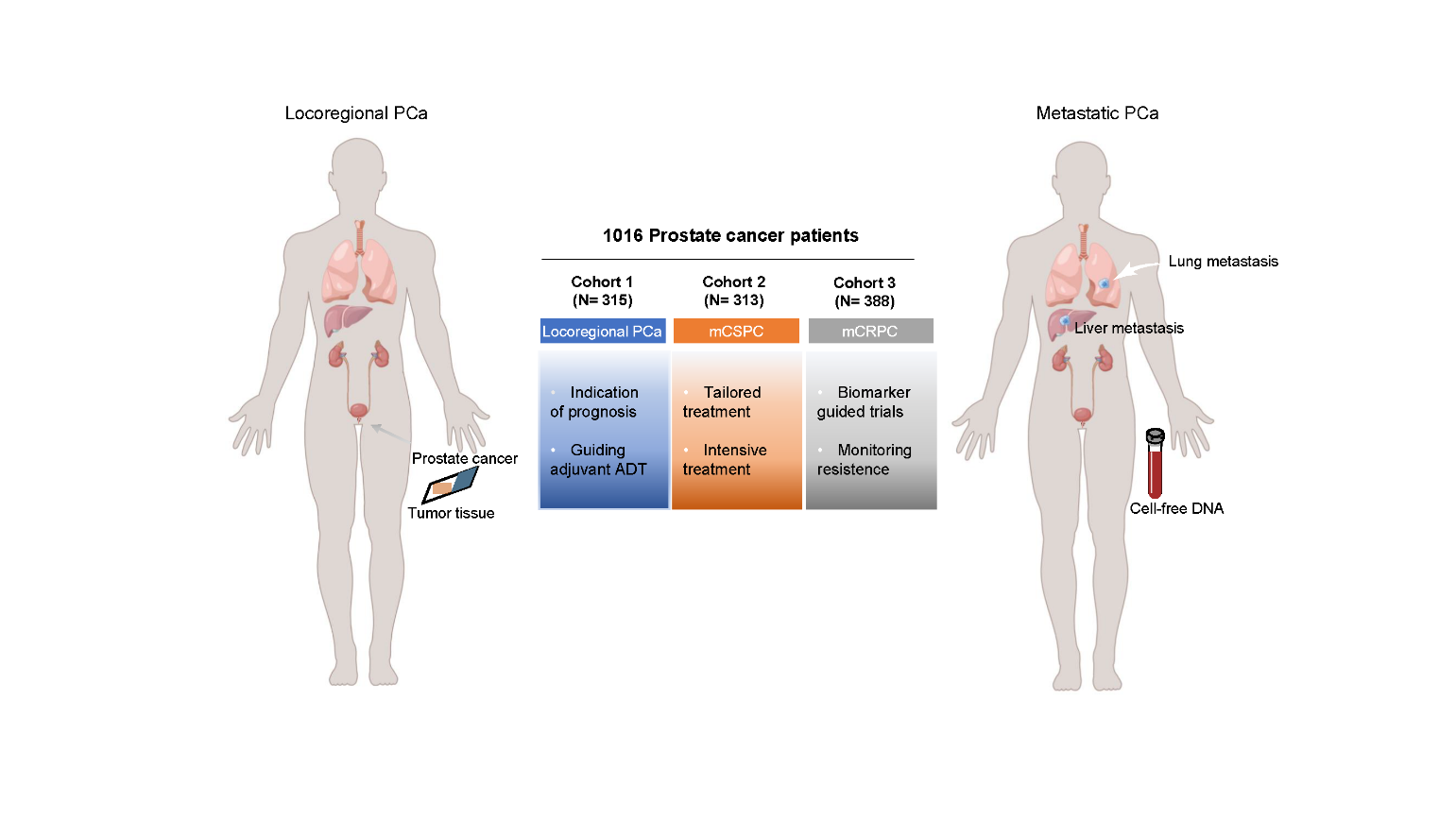


**Supplementary Figure 3. Mutational profile of 41 selected genes in patients in FUSCC-PC cohort classified by treatment and annotated with the variation type and mutation frequency.** The mutation counts in each sample and each gene are provided above and on the right side, respectively.


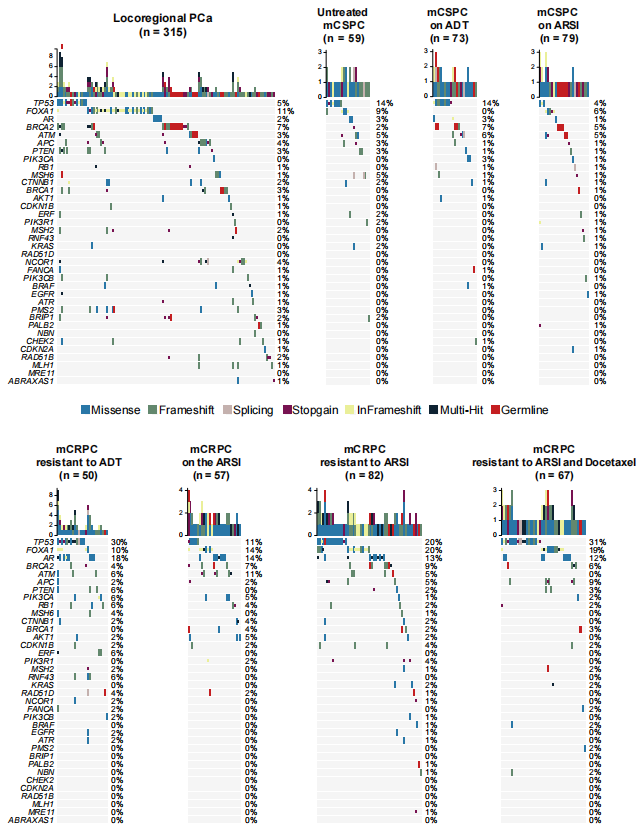


**Supplementary Figure 4. Mutational profile in localized PC (A), mCSPC (B), mCRPC (C)**. Genes are ordered by mutation frequency, and mutations are stratified by mutation types.


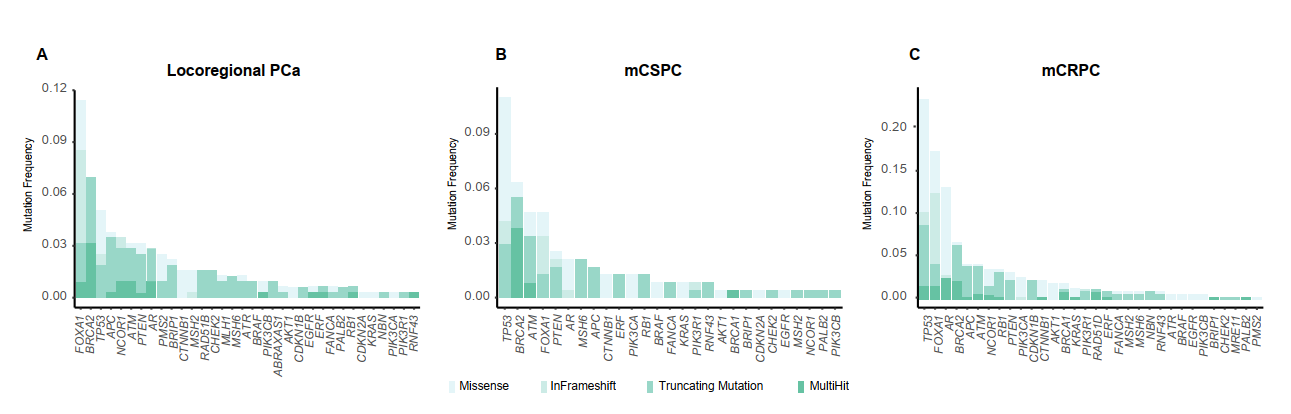


**Supplementary Figure 5. The levels of actionable mutations according to the OncoKB database.**


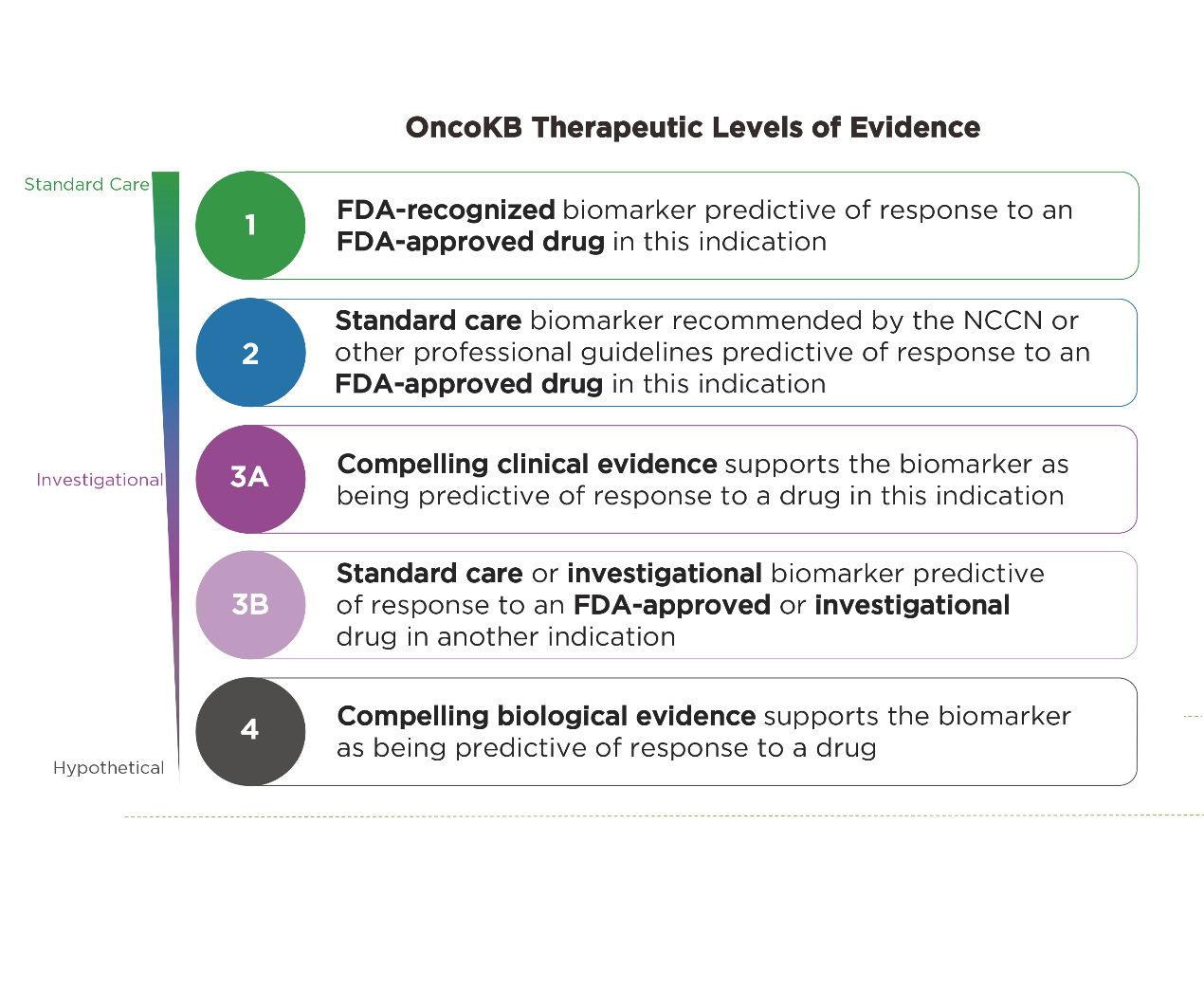


**Supplementary Figure 6. Distribution of Variant allele frequency in the genes with mutation frequency ≥ 1% (A) and split by disease stages (B).**


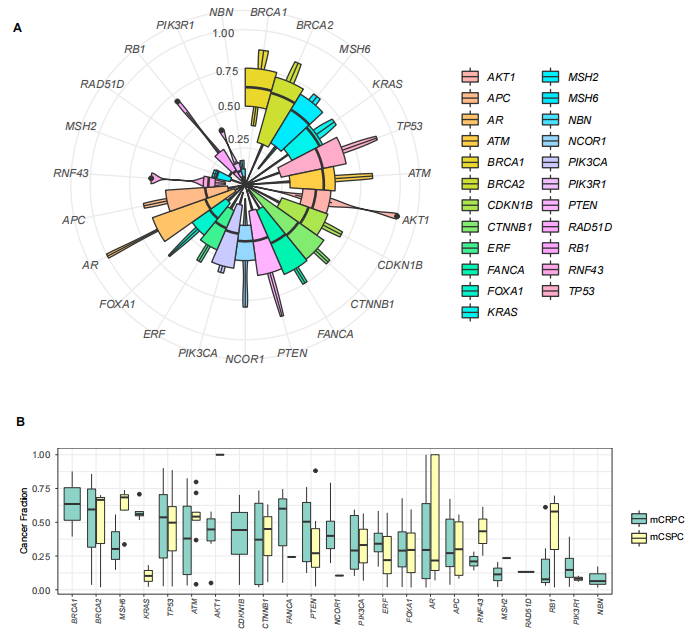


**Supplementary Figure 7. Distribution of participants from different ancestries in the previous genomic studies using a unified sequencing platform.**


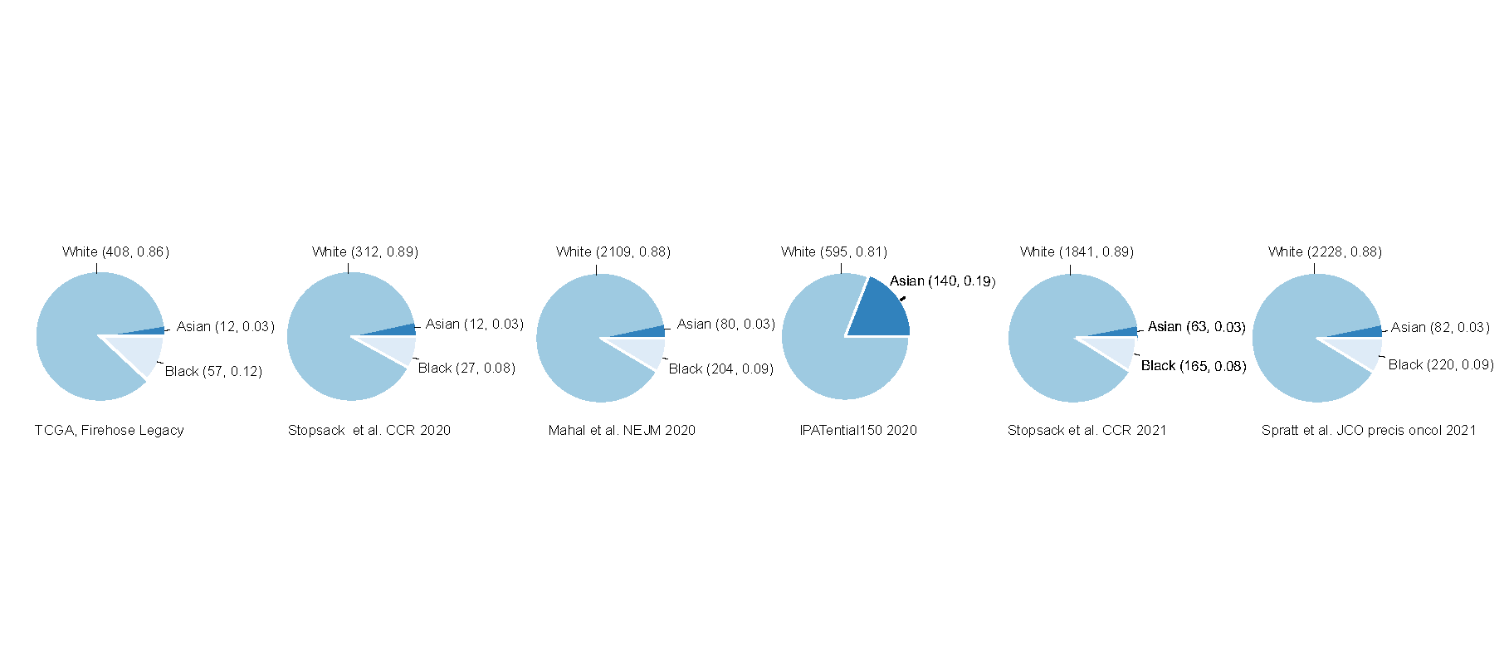


**Supplementary Figure 8. Mutation frequencies across stages and races.**


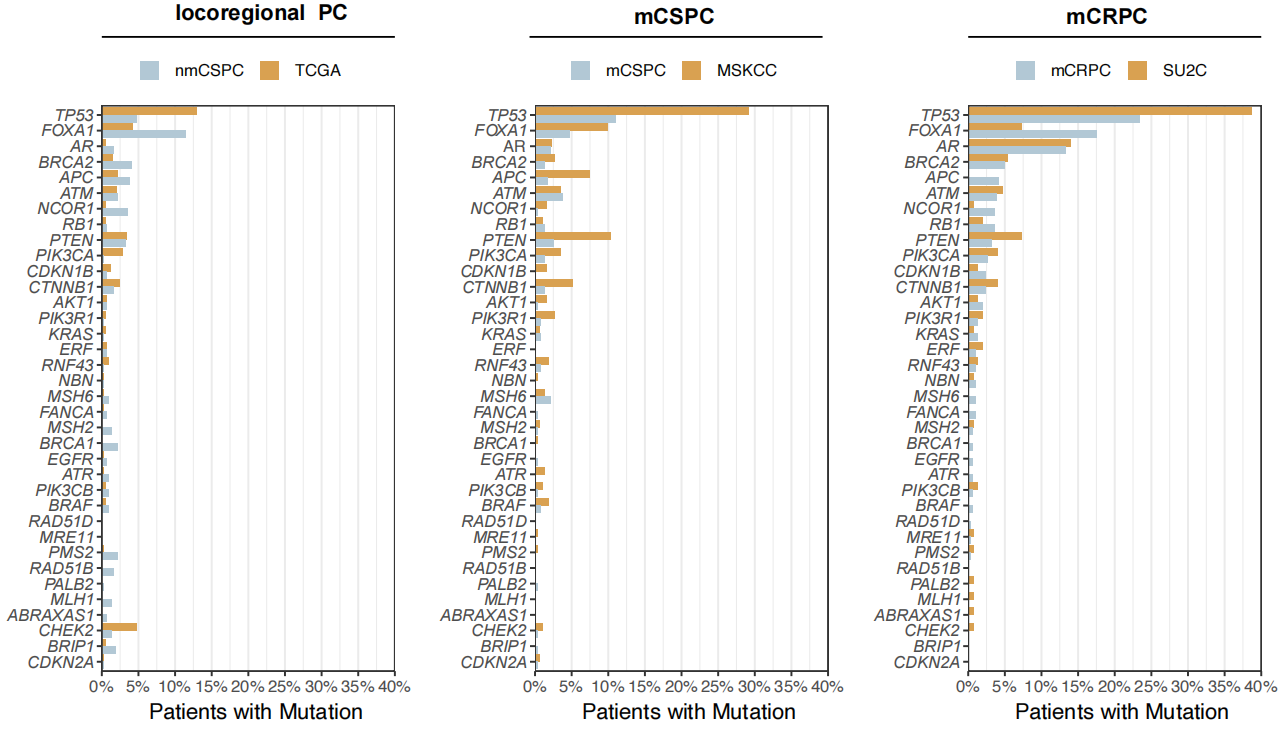


**Supplementary Figure 9. Comparison of genomic mutations between FUSCC-PC cohort 3 with Renji cohort.**


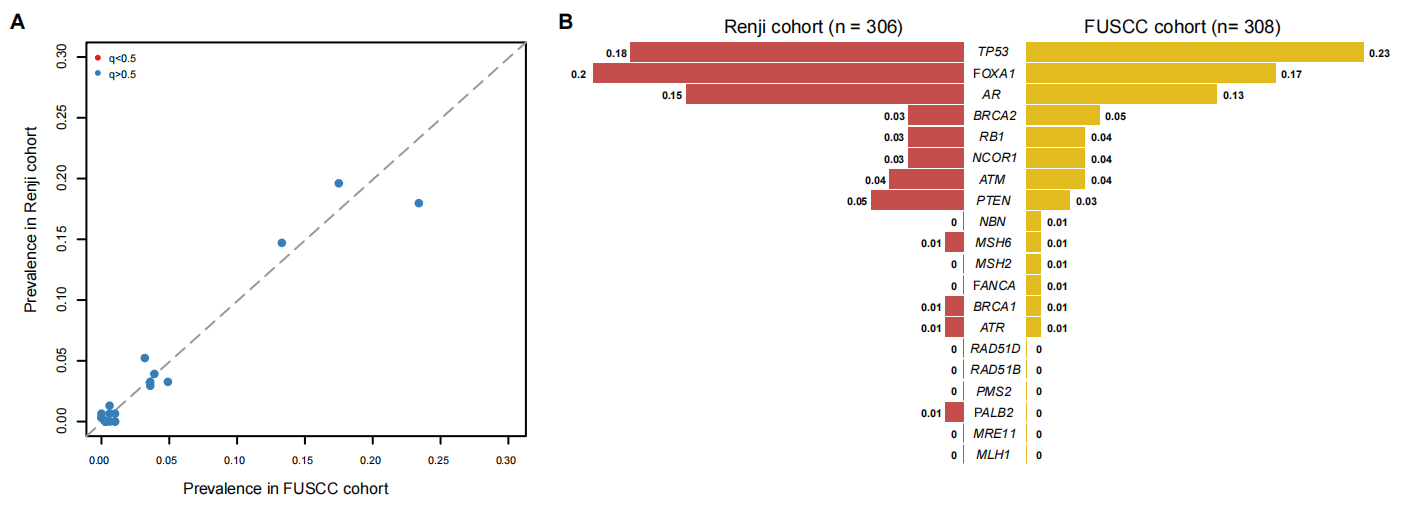


**Supplementary Figure 10. Comparison of genomic mutations between FUSCC-PC cohort 3 with Japanese cohort.**


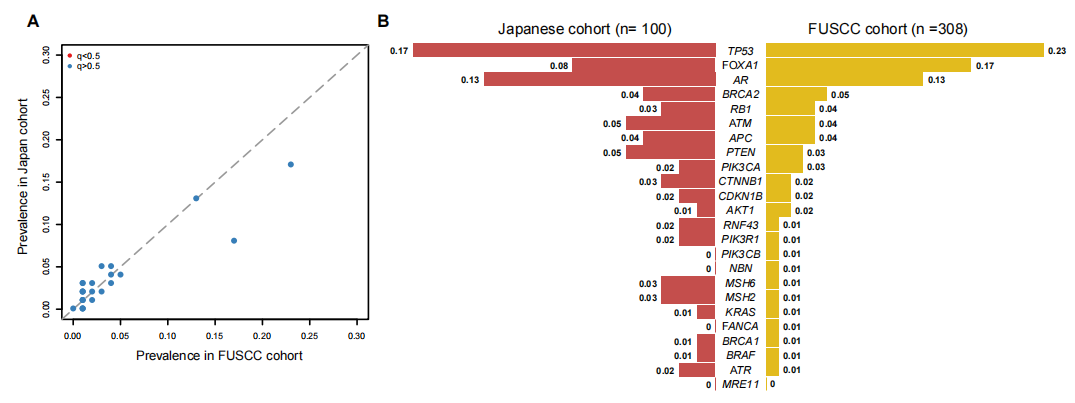


**Supplementary Figure 11. Mutational profile of 41 selected genes in a subset of patients classified by treatment and annotated with the variation type and mutation frequency.** The mutation counts in each sample and each gene are provided above and on the right side, respectively.


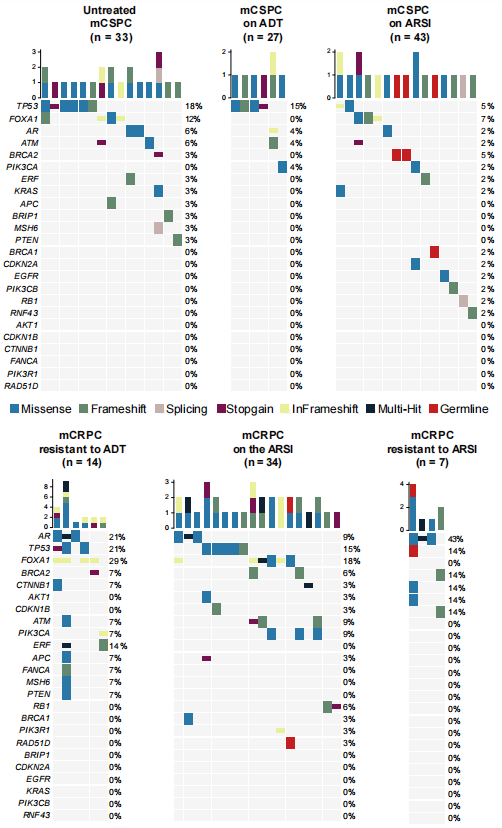


**Supplementary Figure 12. Univariate Cox proportional hazards regression results of overall survival with first-line ARSI in mCRPC patients (n=82).**


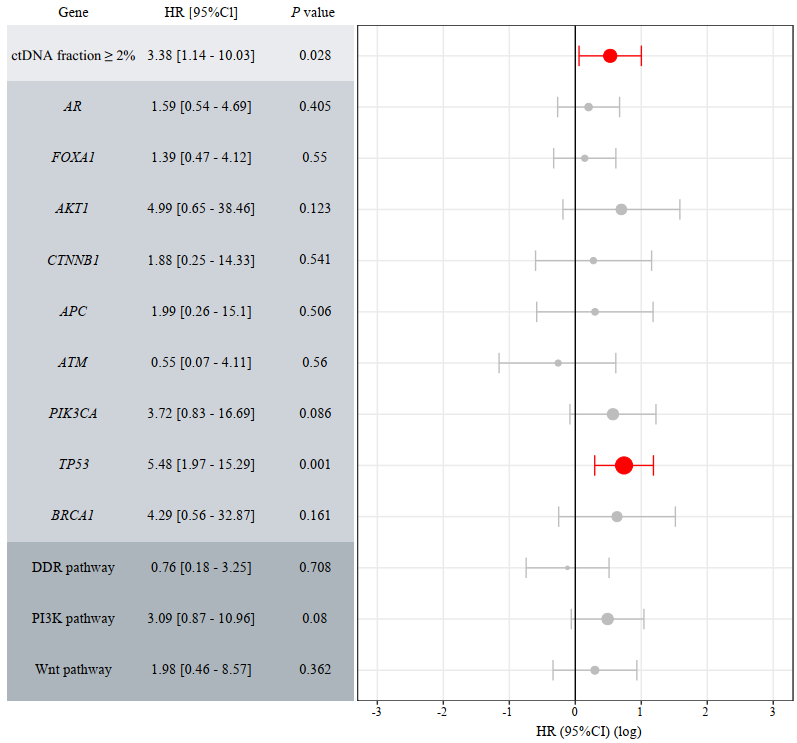

Supplement: Supplementary file 1 — Fig. S1. Validation of variants detected by ctDNA sequencing. Fig. S2. Cohort summary and sample distribution. Fig. S3. Mutational profile of 41 selected genes in patients in FUSCC‐PC cohort classified by treatment and annotated with the variation type and mutation frequency. Fig. S4. Mutational profile in localized PC. Fig. S5. Levels of actionable mutations according to the OncoKB database. Fig. S6. Distribution of Variant allele frequency in the genes with mutation frequency ≥1% (A) and split by disease stages (B). Fig. S7. Distribution of participants from different ancestries in the previous genomic studies using a unified sequencing platform. Fig. S8. Mutation frequencies across stages and races. Fig. S9. Comparison of genomic mutations between FUSCC‐PC cohort 3 with Renji cohort. Fig. S10. Comparison of genomic mutations between FUSCC‐PC cohort 3 with Japanese cohort. Fig. S11. Mutational profile of 41 selected genes in a subset of patients classified by treatment and annotated with the variation type and mutation frequency. Fig. S12. Univariate Cox proportional hazards regression results of overall survival with first‐line ARSI in mCRPC patients (n = 82). [file MOL2-17-2183-s001.docx]
